# Association Between Mineral Metabolism Abnormalities and Vascular Access Complications Among Maintenance Hemodialysis Patients in Ethiopia

**DOI:** 10.1155/ijne/4669522

**Published:** 2026-09-12

**Authors:** Daniel Rebuma Bekele, Tsegay Berhane Gebregiorgis, Alemayehu Abebe Guji, Kedir Negesso Tukeni

**Affiliations:** ^1^ Department of Internal Medicine, Jimma University, Jimma, Ethiopia, ju.edu.et; ^2^ Department of Internal Medicine, Saint Paul Millennium Medical College, Addis Ababa, Ethiopia, sphmmc.edu.et

**Keywords:** CKD-MBD, Ethiopia, hemodialysis, hyperphosphatemia, mineral metabolism, secondary hyperparathyroidism, vascular access

## Abstract

**Background:**

Chronic kidney disease–mineral and bone disorder (CKD‐MBD) contributes to vascular calcification and cardiovascular morbidity among maintenance hemodialysis patients. Mineral metabolism abnormalities, particularly hyperphosphatemia and secondary hyperparathyroidism, may adversely affect vascular access patency and increase vascular access–related complications. However, data from sub‐Saharan Africa remain limited. This study evaluated the association between abnormalities in mineral metabolism and vascular access complications among maintenance hemodialysis patients in Ethiopia.

**Methods:**

A facility‐based cross‐sectional study was conducted among 302 maintenance hemodialysis patients in Addis Ababa, Ethiopia. Sociodemographic, clinical, and laboratory data were collected using structured questionnaires and medical record review. Mineral metabolism parameters included serum calcium, serum phosphorus, and intact parathyroid hormone (iPTH) levels. Vascular access complications were assessed clinically and from dialysis records. Multivariable logistic regression analysis was performed to identify factors independently associated with vascular access complications.

**Results:**

The mean age of participants was 52.6 ± 14.2 years, and 59.0% were male. Vascular access complications were identified in 72 (23.8%) participants. Hyperphosphatemia, hypocalcemia, and elevated iPTH were present in 28.0%, 27.7%, and 47.4% of patients, respectively. Patients with vascular access complications had significantly higher mean serum phosphorus levels and iPTH concentrations compared with those without complications (*p* < 0.05). In multivariable analysis, hyperphosphatemia (adjusted odds ratio [AOR] = 2.41, 95% CI: 1.32–4.39), elevated iPTH (AOR = 2.87, 95% CI: 1.58–5.21), and dialysis duration > 24 months (AOR = 1.94, 95% CI: 1.08–3.47) were independently associated with vascular access complications.

**Conclusions:**

Abnormalities in mineral metabolism were significantly associated with vascular access complications among maintenance hemodialysis patients in Ethiopia. Hyperphosphatemia and elevated iPTH emerged as important independent predictors of vascular access dysfunction. Strengthening CKD‐MBD management and optimizing mineral metabolism control may help reduce vascular access complications and improve dialysis outcomes in resource‐limited settings.

## 1. Introduction

Vascular access remains the cornerstone of effective maintenance hemodialysis therapy, and preservation of vascular access function is essential for adequate dialysis delivery and improved patient outcomes. However, vascular access complications, including thrombosis, stenosis, infection, aneurysm formation, and access failure, remain major causes of morbidity, hospitalization, and healthcare utilization among patients receiving chronic dialysis [1, 2]. Recurrent vascular access dysfunction may lead to interrupted dialysis treatment, repeated invasive procedures, increased healthcare costs, and reduced survival.

Chronic kidney disease–mineral and bone disorder (CKD‐MBD) is a systemic complication of chronic kidney disease characterized by abnormalities in calcium, phosphorus, parathyroid hormone, and vitamin D metabolism, as well as extraskeletal calcification and bone disease [3]. Disturbances in mineral metabolism become increasingly prevalent with progression to end‐stage kidney disease (ESKD) and are highly common among maintenance hemodialysis patients [4]. Persistent hyperphosphatemia and secondary hyperparathyroidism contribute to vascular smooth muscle cell transformation, medial vascular calcification, endothelial dysfunction, and arterial stiffness [5, 6].

Growing evidence suggests that CKD‐MBD may also adversely affect vascular access outcomes in hemodialysis patients. Mineral metabolism abnormalities have been associated with vascular calcification involving both native vessels and dialysis access sites, potentially increasing the risk of access stenosis, thrombosis, and vascular dysfunction [7, 8]. Elevated serum phosphorus and intact parathyroid hormone (iPTH) levels have been linked to accelerated vascular calcification and reduced vascular compliance, factors that may compromise arteriovenous fistula maturation and long‐term vascular access [9, 10].

Several studies from high‐income countries have demonstrated associations between hyperphosphatemia, secondary hyperparathyroidism, and adverse vascular outcomes among dialysis patients [11, 12]. However, evidence from low‐ and middle‐income countries, particularly sub‐Saharan Africa, remains scarce. In many African dialysis settings, limited access to phosphate binders, calcimimetics, active vitamin D analogs, and regular biochemical monitoring may contribute to poor CKD‐MBD control [13, 14]. These healthcare limitations may increase the burden of vascular access complications and negatively affect dialysis outcomes.

In Ethiopia, the number of patients receiving maintenance hemodialysis has increased substantially over recent years, yet data regarding CKD‐MBD and vascular access outcomes remain limited. Understanding the relationship between mineral metabolism abnormalities and vascular access complications may help guide targeted interventions aimed at improving dialysis care and reducing vascular morbidity.

Therefore, this study aimed to assess the association between abnormalities in mineral metabolism and vascular access complications among maintenance hemodialysis patients in Ethiopia.

## 2. Methods

### 2.1. Study Design and Setting

An institution‐based cross‐sectional study was conducted among adult patients receiving maintenance hemodialysis at St Paul’s Hospital Millennium Medical College (SPHMMC) and affiliated private dialysis centers in Addis Ababa, Ethiopia, between September 2025 and February 2026. SPHMMC is one of the major tertiary referral hospitals in Ethiopia and provides nephrology and dialysis services for patients with ESKD. In addition to patients receiving dialysis at SPHMMC, the study included patients undergoing hemodialysis at private dialysis centers while attending routine nephrology follow‐up at SPHMMC.

### 2.2. Study Population

The study population consisted of adult patients with ESKD receiving maintenance hemodialysis during the study period.

### 2.3. Inclusion Criteria

Participants were included if they were aged 18 years or older, had been receiving maintenance hemodialysis for at least 3 months, attended regular follow‐up at SPHMMC or affiliated private dialysis centers, and provided written informed consent.

### 2.4. Exclusion Criteria

Patients were excluded if they had acute kidney injury requiring temporary dialysis, had been receiving dialysis for less than 3 months, were critically ill during data collection, had cognitive or communication impairments that prevented reliable participation in interviews, or declined to participate in the study.

### 2.5. Sample Size Determination and Sampling Procedure

The sample size was calculated using the single population proportion formula:

*n* = *Z* ^2^ *p*(1 − *p*)/*d* ^2^ where

• *n* = minimum required sample size;
• *Z* = standard normal deviate at 95% confidence interval (1.96);
• *p* = assumed prevalence of CKD‐MBD abnormalities among maintenance hemodialysis patients;
• *d* = margin of error.

Because no prior Ethiopian study had comprehensively assessed CKD‐MBD prevalence among maintenance hemodialysis patients, a conservative prevalence estimate of 50% (*p* = 0.5) was used to maximize sample size, with a margin of error of 5%. After adjustment for the finite study population and anticipated nonresponse, the final target sample size was determined. Consecutive sampling was used to recruit eligible participants attending dialysis sessions or nephrology follow‐up during the study period until the required sample size was achieved. A total of 339 patients were screened for eligibility. Thirty‐seven patients were excluded because of acute illness, dialysis duration less than 3 months, cognitive impairment, or refusal to participate. Ultimately, 302 participants were included in the final analysis.

### 2.6. Data Collection Procedures

Data were collected through structured face‐to‐face interviews and medical record review using a standardized questionnaire developed from previous CKD‐MBD studies and Kidney Disease: Improving Global Outcomes (KDIGO) recommendations. The questionnaire captured sociodemographic characteristics, dialysis history, CKD‐MBD–related symptoms and complications, medication use, and quality‐of‐life information. Laboratory and clinical data were abstracted from medical records.

Data collection was conducted by trained physicians and nephrology staff under close supervision. The questionnaire was pretested before implementation, and necessary modifications were made to improve clarity and consistency.

### 2.7. Operational Definitions

• Hyperphosphatemia: serum phosphorus > 5.5 mg/dL.
• Hypocalcemia: corrected serum calcium < 8.4 mg/dL.
• Elevated iPTH: serum iPTH > 300 pg/mL.
• CKD‐MBD–related complications: included bone pain, pruritus, fractures, cardiovascular complications, and vascular access complications. Bone pain was defined as persistent musculoskeletal pain reported during patient interviews or documented in medical records. Pruritus was defined as recurrent generalized itching not attributable to primary dermatologic conditions.
• Vascular access complications were defined as the presence of clinically documented vascular access dysfunction, including thrombosis, stenosis, recurrent access failure, aneurysm formation, infection, or need for vascular access intervention during maintenance hemodialysis follow‐up. Vascular access complications included recurrent thrombosis, stenosis, infection, access failure, or repeated vascular access interventions.

### 2.8. Quality‐of‐Life Assessment

Quality of life was assessed using a dialysis‐specific quality‐of‐life instrument adapted for local use. The instrument evaluated physical, emotional, and social domains of well‐being. Higher scores indicated better quality of life.

### 2.9. Laboratory Measurements

Laboratory data were abstracted from the most recent biochemical investigations performed within the preceding 3 months. Laboratory parameters included serum calcium, serum phosphorus, iPTH, and serum 25‐hydroxyvitamin D levels. Laboratory results were interpreted according to KDIGO‐aligned thresholds and local laboratory reference ranges.

### 2.10. Data Quality Assurance

Data collectors received standardized training before data collection. The questionnaire was pretested before implementation. Completed questionnaires were reviewed daily for completeness and consistency. Data entry included range and consistency checks, and random verification was performed to minimize transcription errors.

### 2.11. Data Processing and Statistical Analysis

Data were entered into EpiData and exported to SPSS version 27 for analysis. Continuous variables were summarized using mean ± standard deviation or median with interquartile range (IQR), depending on data distribution. Categorical variables were presented as frequencies and percentages. Comparisons of quality‐of‐life scores across CKD‐MBD complication groups were performed using independent *t*‐tests, one‐way analysis of variance (ANOVA), or nonparametric equivalents as appropriate. Bivariate analyses were conducted to identify variables associated with reduced quality of life.

Multiple linear regression analysis was performed to identify independent predictors of total quality‐of‐life scores. Variables with *p* values < 0.20 in bivariate analysis and clinically relevant variables identified from previous literature were entered into the multivariable model. Regression coefficients (*β*) with 95% confidence intervals (CIs) were reported. Model assumptions, including normality, linearity, homoscedasticity, and multicollinearity, were assessed before final analysis. Statistical significance was considered at *p* < 0.05.

### 2.12. Ethical Considerations

Ethical approval was obtained from the Institutional Review Board of SPHMMC. Written informed consent was obtained from all participants before enrollment. Participation was voluntary, and confidentiality was maintained throughout the study using anonymized study identification numbers. All collected data were used solely for research purposes.

## 3. Results

### 3.1. Participant Characteristics

A total of 302 maintenance hemodialysis patients were included in the study. The mean age of participants was 52.6 ± 14.2 years, and 178 (59.0%) were male. More than half of the participants had been receiving hemodialysis for longer than 24 months. Hypertension was the most common comorbidity, followed by diabetes mellitus.

The mean serum calcium level was 8.9 ± 0.8 mg/dL, while the mean serum phosphorus level was 5.2 ± 1.1 mg/dL. The median iPTH level was 310 pg/mL (interquartile range [IQR]: 220–420 pg/mL). Hyperphosphatemia, hypocalcemia, and elevated iPTH were identified in 28.0%, 27.7%, and 47.4% of participants, respectively (Table 1).

**TABLE 1:** Sociodemographic, clinical, and mineral metabolism characteristics of maintenance hemodialysis patients (*N* = 302).

Variable	Value
Age, years (mean ± SD)	52.6 ± 14.2
Age ≥ 60 years, *n* (%)	118 (39.1)
Male sex, *n* (%)	178 (59.0)
Dialysis duration, months (median [IQR])	36 [24–60]
Dialysis duration > 24 months, *n* (%)	196 (64.9)
Hypertension, *n* (%)	246 (81.5)
Diabetes mellitus, *n* (%)	112 (37.1)
Arteriovenous fistula as vascular access, *n* (%)	214 (70.9)
Central venous catheter use, *n* (%)	88 (29.1)
Serum calcium (mg/dL) (mean ± SD)	8.9 ± 0.8
Serum phosphorus (mg/dL) (mean ± SD)	5.2 ± 1.1
Intact parathyroid hormone (iPTH) (pg/mL) (median [IQR])	310 [220–420]
Hyperphosphatemia, *n* (%)	83 (28.0)
Hypocalcemia, *n* (%)	82 (27.7)
Elevated iPTH, *n* (%)	143 (47.4)
Vitamin D deficiency, *n* (%)	59 (19.5)
Vascular access complications, *n* (%)	72 (23.8)

*Note:* Values are presented as mean ± standard deviation (SD), median (interquartile range [IQR]), or frequency (%). Hyperphosphatemia was defined as serum phosphorus > 5.5 mg/dL. Hypocalcemia was defined as corrected serum calcium < 8.4 mg/dL. Elevated intact parathyroid hormone (iPTH) was defined as iPTH > 300 pg/mL. IQR, interquartile range.

Abbreviations: CKD‐MBD, chronic kidney disease–mineral and bone disorder; SD, standard deviation.

### 3.2. Prevalence and Patterns of Vascular Access Complications

Vascular access complications were identified in 72 (23.8%) participants. The most common vascular access complications included recurrent thrombosis, stenosis requiring intervention, vascular access infection, and repeated vascular access failure.

**FIGURE 1:**
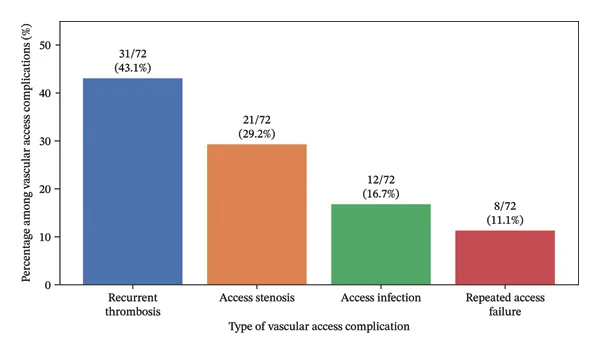
Distribution of vascular access complications among maintenance hemodialysis patients. Bar chart illustrating the frequency and percentage distribution of vascular access complications among maintenance hemodialysis patients with vascular access dysfunction. Recurrent thrombosis was the most common complication, followed by access stenosis, access infection, and repeated access failure.

Among participants with vascular access complications, recurrent thrombosis was the most frequently documented complication, followed by access stenosis and access‐related infections. Patients with vascular access complications had longer dialysis duration and less favorable mineral metabolism profiles compared with those without complications (Figure 1).

### 3.3. Mineral Metabolism Abnormalities According to Vascular Access Status

Patients with vascular access complications had significantly higher serum phosphorus and iPTH levels compared with patients without vascular access complications. The mean serum phosphorus level among participants with vascular access complications was 5.9 ± 1.2 mg/dL compared with 4.9 ± 1.0 mg/dL among participants without complications (*p* < 0.001). Similarly, median iPTH levels were substantially higher among participants with vascular access complications than among those without complications. Corrected serum calcium levels were lower among participants with vascular access complications, although the difference was less pronounced.

**FIGURE 2:**
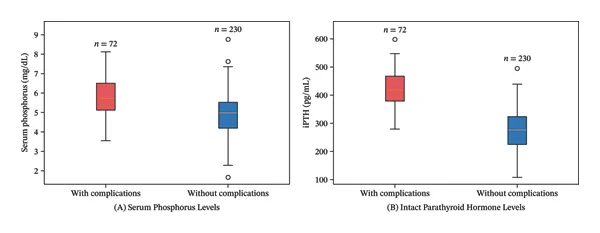
Distribution of serum phosphorus and intact parathyroid hormone (iPTH) levels according to vascular access complication status. Boxplots comparing serum phosphorus and intact parathyroid hormone (iPTH) levels between patients with and without vascular access complications. Patients with vascular access complications demonstrated significantly higher serum phosphorus and iPTH levels.

The prevalence of hyperphosphatemia and elevated iPTH was significantly greater among participants with vascular access complications compared with those without complications (Table 2 and Figure 2).

**TABLE 2:** Mineral metabolism abnormalities according to vascular access complication status among maintenance hemodialysis patients (*N* = 302).

Variable	With vascular access complications (*n* = 72)	Without vascular access complications (*n* = 230)	*p* value
Serum calcium (mg/dL) (mean ± SD)	8.5 ± 0.7	9.0 ± 0.8	0.021
Serum phosphorus (mg/dL) (mean ± SD)	5.9 ± 1.2	4.9 ± 1.0	< 0.001
Intact parathyroid hormone (iPTH) (pg/mL) (median [IQR])	410 [320–540]	280 [210–360]	< 0.001
Hyperphosphatemia, *n* (%)	38 (52.8)	45 (19.6)	< 0.001
Hypocalcemia, *n* (%)	29 (40.3)	53 (23.0)	0.006
Elevated iPTH, *n* (%)	49 (68.1)	94 (40.9)	< 0.001
Vitamin D deficiency, *n* (%)	21 (29.2)	38 (16.5)	0.018
Dialysis duration > 24 months, *n* (%)	57 (79.2)	139 (60.4)	0.004
Arteriovenous fistula use, *n* (%)	42 (58.3)	172 (74.8)	0.009
Central venous catheter use, *n* (%)	30 (41.7)	58 (25.2)	0.009

*Note:* Values are presented as mean ± standard deviation (SD), median [interquartile range (IQR)], or frequency (%). *p* values were calculated using the independent samples *t*‐test, Mann–Whitney *U* test, chi‐square test, or Fisher’s exact test as appropriate. Hyperphosphatemia was defined as serum phosphorus > 5.5 mg/dL. Hypocalcemia was defined as corrected serum calcium < 8.4 mg/dL. Elevated intact parathyroid hormone (iPTH) was defined as iPTH > 300 pg/mL. AVF, arteriovenous fistula; IQR, interquartile range.

Abbreviations: CKD‐MBD, chronic kidney disease–mineral and bone disorder; SD, standard deviation.

### 3.4. Association Between Mineral Metabolism Abnormalities and Vascular Access Complications

In bivariable logistic regression analysis, dialysis duration > 24 months, hyperphosphatemia, hypocalcemia, and elevated iPTH were significantly associated with vascular access complications. Diabetes mellitus and older age also demonstrated positive associations but did not remain statistically significant after adjustment.

In multivariable logistic regression analysis, hyperphosphatemia remained independently associated with vascular access complications (adjusted odds ratio [AOR] = 2.41, 95% confidence interval [CI]: 1.32–4.39; *p* = 0.004). Elevated iPTH was also independently associated with vascular access complications (AOR = 2.87, 95% CI: 1.58–5.21; *p* < 0.001).

Patients receiving maintenance hemodialysis for more than 24 months had nearly twofold higher odds of vascular access complications compared with those receiving dialysis for shorter durations (AOR = 1.94, 95% CI: 1.08–3.47; *p* = 0.026). Hypocalcemia demonstrated a positive but borderline association with vascular access complications after adjustment (Table 3).

**TABLE 3:** Multivariable logistic regression analysis of factors associated with vascular access complications among maintenance hemodialysis patients (*N* = 302).

Variable	Crude odds ratio (COR)	Adjusted odds ratio (AOR)	95% confidence interval	*p* value
Age ≥ 60 years	1.58	1.29	0.72–2.31	0.392
Female sex	1.24	1.11	0.62–1.99	0.724
Dialysis duration > 24 months	2.49	1.94	1.08–3.47	0.026
Diabetes mellitus	1.71	1.39	0.78–2.49	0.264
Hyperphosphatemia	4.58	2.41	1.32–4.39	0.004
Hypocalcemia	2.26	1.69	0.92–3.12	0.089
Elevated intact parathyroid hormone (iPTH)	3.08	2.87	1.58–5.21	< 0.001
Vitamin D deficiency	2.08	1.54	0.79–3.02	0.201
Central venous catheter use	2.13	1.72	0.95–3.13	0.074

*Note:* Model statistics: Hosmer–Lemeshow goodness‐of‐fit test *p* value = 0.61, Nagelkerke *R* ^2^ = 0.34, Overall model *p* value < 0.001. Multivariable logistic regression analysis was performed using vascular access complication status as the dependent variable. Variables with *p* < 0.20 in bivariable analysis were included in the multivariable model. Hyperphosphatemia was defined as serum phosphorus > 5.5 mg/dL. Hypocalcemia was defined as corrected serum calcium < 8.4 mg/dL. Elevated intact parathyroid hormone (iPTH) was defined as iPTH > 300 pg/mL. iPTH, intact parathyroid hormone.

Abbreviations: AOR, adjusted odds ratio; CI, confidence interval; COR, crude odds ratio.

### 3.5. Relationship Between Mineral Metabolism Markers and Vascular Access Complications

Scatterplot and boxplot analyses demonstrated progressively increasing rates of vascular access complications with worsening serum phosphorus and iPTH levels. Participants with elevated phosphorus and markedly elevated iPTH concentrations showed the highest burden of vascular access dysfunction.

**FIGURE 3:**
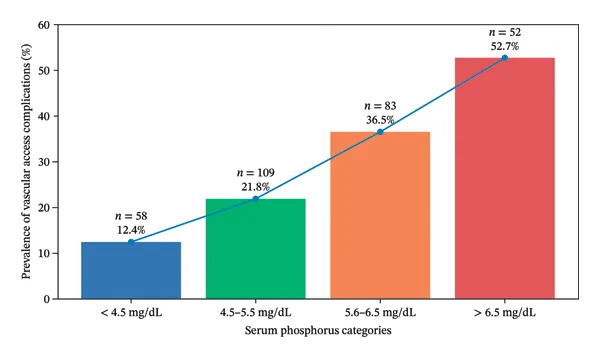
Increasing prevalence of vascular access complications across serum phosphorus categories. Bar and trend plot demonstrating the progressive increase in vascular access complication prevalence across higher serum phosphorus categories among maintenance hemodialysis patients, suggesting a dose–response relationship between worsening phosphorus control and vascular morbidity.

**FIGURE 4:**
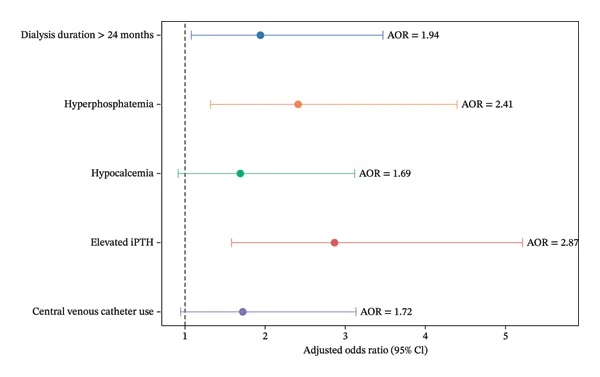
Forest plot of factors independently associated with vascular access complications. Forest plot illustrating adjusted odds ratios (AORs) and 95% confidence intervals for independent predictors of vascular access complications identified in multivariable logistic regression analysis. Hyperphosphatemia, elevated intact parathyroid hormone levels, and prolonged dialysis duration were independently associated with vascular access complications.

The prevalence of vascular access complications increased across progressively higher phosphorus categories, suggesting a dose–response relationship between worsening mineral metabolism abnormalities and vascular morbidity (Figures 3 and 4).

**FIGURE 5:**
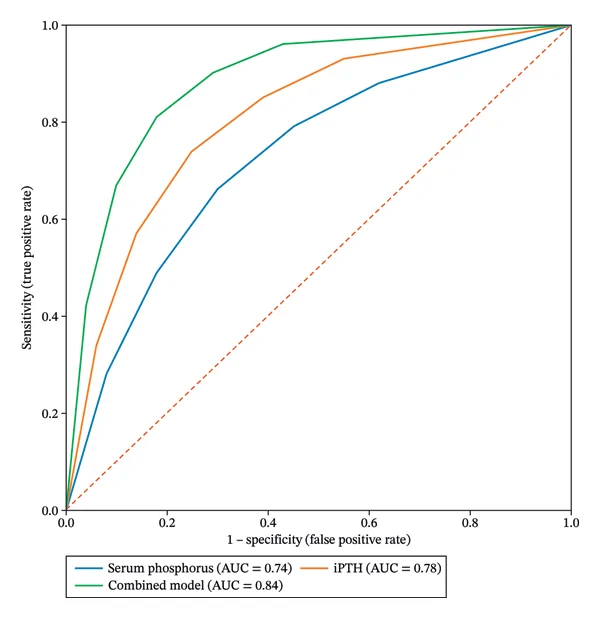
Receiver operating characteristic (ROC) curves for prediction of vascular access complications.

Receiver operating characteristic (ROC) curves demonstrating the predictive performance of serum phosphorus, iPTH, and the combined multivariable model for vascular access complications among maintenance hemodialysis patients. The combined model demonstrated the highest discriminatory ability (AUC = 0.84), followed by iPTH (AUC = 0.78) and serum phosphorus (AUC = 0.74), indicating good predictive accuracy of mineral metabolism abnormalities for vascular access dysfunction (Figure 5).

## 4. Discussion

This study evaluated the association between mineral metabolism abnormalities and vascular access complications among maintenance hemodialysis patients in Ethiopia (Graphical Abstract). The findings demonstrated a substantial burden of vascular access complications, with nearly one‐quarter of participants experiencing clinically significant vascular access dysfunction. Hyperphosphatemia, elevated iPTH, and prolonged dialysis duration were independently associated with vascular access complications. Patients with vascular access complications also had significantly higher serum phosphorus and iPTH levels compared with those without complications, supporting the important role of CKD‐MBD in vascular morbidity among hemodialysis patients.

The prevalence of vascular access complications observed in this study is comparable to findings from previous hemodialysis cohorts, where vascular access dysfunction remains a major contributor to morbidity, hospitalization, and interrupted dialysis [1, 2]. Recurrent thrombosis and access stenosis were the most common complications identified, consistent with reports from international dialysis registries and observational studies [3, 4]. Vascular access dysfunction is clinically significant because it may compromise dialysis adequacy, increase procedural interventions, and negatively affect long‐term patient outcomes.

One of the principal findings of this study was the strong association between hyperphosphatemia and vascular access complications. Patients with elevated serum phosphorus levels had more than twofold higher odds of vascular access complications after adjustment for potential confounders. Hyperphosphatemia is a well‐established promoter of vascular calcification in chronic kidney disease [5, 6]. Elevated phosphorus concentrations induce osteogenic transformation of vascular smooth muscle cells, promote medial arterial calcification, and increase vascular stiffness [7]. These pathological changes may impair vascular elasticity and compromise vascular access patency, particularly in arteriovenous fistulas exposed to repeated hemodynamic stress.

The progressive increase in vascular access complication prevalence across higher serum phosphorus categories observed in this study further supports a dose–response relationship between worsening mineral metabolism abnormalities and vascular morbidity. Similar associations between phosphorus abnormalities and vascular calcification have been described in maintenance hemodialysis populations from Europe and North America [8, 9]. However, evidence from sub‐Saharan Africa remains scarce. The present study, therefore, contributes important regional evidence regarding the vascular consequences of CKD‐MBD among African dialysis populations.

Elevated iPTH was another independent predictor of vascular access complications in this study. Secondary hyperparathyroidism contributes to high‐turnover bone disease and may accelerate vascular calcification through persistent disturbances in calcium–phosphorus homeostasis [10, 11]. Clinical studies have shown that severe secondary hyperparathyroidism is associated with endothelial dysfunction, arterial stiffness, and increased cardiovascular calcification burden [13]. These vascular alterations may adversely affect both native blood vessels and dialysis access sites, increasing susceptibility to stenosis, thrombosis, and recurrent vascular access failure.

The observed association between prolonged dialysis duration and vascular access complications is also biologically plausible. Patients receiving long‐term hemodialysis are exposed to chronic vascular injury, repeated cannulation, persistent inflammation, and cumulative mineral metabolism abnormalities, all of which may contribute to progressive vascular damage and access dysfunction [14]. Longer dialysis duration may additionally reflect greater cumulative exposure to hyperphosphatemia and secondary hyperparathyroidism.

Hypocalcemia demonstrated a positive but borderline association with vascular access complications after multivariable adjustment. Although serum calcium abnormalities are recognized components of CKD‐MBD, the relationship between calcium levels and vascular outcomes is often complex and influenced by concurrent phosphorus abnormalities, vitamin D status, and parathyroid hormone activity [15]. The lack of a strong independent association in the present study may reflect the multifactorial nature of vascular access dysfunction.

The findings of this study have important clinical implications for dialysis care in resource‐limited settings. In many sub‐Saharan African countries, including Ethiopia, access to phosphate binders, calcimimetics, active vitamin D analogs, and regular CKD‐MBD monitoring remains limited [16, 17]. Financial constraints may further reduce adherence to dietary phosphorus restriction and prescribed medications. Consequently, many patients experience persistent mineral metabolism abnormalities that may increase vascular and cardiovascular complications.

Routine monitoring and early management of hyperphosphatemia and secondary hyperparathyroidism may therefore play an important role in preserving vascular access function and improving dialysis outcomes. Integrating CKD‐MBD management into standard vascular access surveillance protocols may help identify high‐risk patients earlier and reduce the burden of access‐related morbidity.

This study has several strengths. It evaluated clinically relevant vascular access outcomes alongside biochemical markers of CKD‐MBD in a relatively large maintenance hemodialysis population. In addition, the study provides important evidence from a resource‐limited African setting where data on CKD‐MBD and vascular access complications remain scarce.

However, several limitations should be acknowledged. First, the cross‐sectional design limits causal inference between mineral metabolism abnormalities and vascular access complications. Second, the study relied partly on routinely collected laboratory data, which may have introduced variability in measurement timing. Third, the study was conducted in dialysis centers within Addis Ababa, potentially limiting generalizability to rural settings or other countries. Finally, residual confounding related to dialysis adequacy, inflammatory markers, nutritional status, medication adherence, and vascular access care practices could not be completely excluded.

## 5. Conclusion

Abnormalities in mineral metabolism were significantly associated with vascular access complications among maintenance hemodialysis patients in Ethiopia. Hyperphosphatemia and elevated iPTH levels emerged as important independent predictors of vascular access dysfunction, while prolonged dialysis duration was also associated with increased vascular access complications. These findings highlight the potential contribution of CKD‐MBD to vascular morbidity among hemodialysis patients and emphasize the importance of routine mineral metabolism monitoring and optimized CKD‐MBD management to preserve vascular access function and improve dialysis outcomes in resource‐limited settings.

## Author Contributions

The paper was conceived and written collaboratively by Kedir Negesso Tukeni, Daniel Rebuma Bekele, and Alemayehu Abebe Guji, with Tsegay Berhane Gebregiorgis taking the lead on the initial draft. The analysis of the cases involved contributions from Kedir Negesso Tukeni, Daniel Rebuma Bekele, and Alemayehu Abebe Guji, while all authors participated in the revision process and approved the final manuscript.

## Funding

The authors received no specific funding for this work.

## Consent

The authors have nothing to report.

## Conflicts of Interest

The authors declare no conflicts of interest.

## Supporting Information

Additional supporting information can be found online in the Supporting Information section.

## Supporting information


**Supporting Information** Graphical abstract. Graphical summary illustrating the relationship between chronic kidney disease–mineral and bone disorder (CKD‐MBD) abnormalities and vascular access complications among maintenance hemodialysis patients in Ethiopia. The figure demonstrates the proposed pathophysiologic pathway linking hyperphosphatemia and elevated parathyroid hormone levels to vascular calcification, vascular stiffness, and vascular access dysfunction.

## Data Availability

The data that support the findings of this study are available from the corresponding author upon reasonable request.
